# Surgery for non-small cell lung cancer in patients with a history of cardiovascular surgery

**DOI:** 10.1007/s00595-016-1386-5

**Published:** 2016-07-21

**Authors:** Hideyuki Maeda, Masato Kanzaki, Kei Sakamoto, Tamami Isaka, Kenji Yamazaki, Takamasa Onuki

**Affiliations:** 1 Department of Surgery I, Tokyo Women’s Medical University, 8-1 Kawada-cho, Shinjuku-ku, Tokyo, 162-8666 Japan; 2 Department of Cardiovascular Surgery, Tokyo Women’s Medical University, Tokyo, Japan

**Keywords:** Lung cancer, Surgery, Cardiovascular surgery

## Abstract

**Purpose:**

To clarify if previous cardiovascular surgery (CVS) affects the postoperative outcome of surgery for non-small cell lung cancer (NSCLC).

**Methods:**

We reviewed, retrospectively, the medical records of 36 patients with a history of CVS, who underwent lung cancer surgery at a single institution (study group; SG) and compared their characteristics and postoperative outcomes with those of patients without a history of CVS history (control group; CG), and also with those of patients with coexisting cardiovascular diseases in the CG (specified control group; SCG). Finally, we used a thoracic revised cardiac risk index (ThRCRI) to evaluate the risk of perioperative cardiovascular events.

**Results:**

There was a significant difference in the ThRCRI classifications between the SG and the SCG (*p* < 0.0001). There were no significant differences in the incidence of intraoperative and postoperative complications between the SG and CG, or between the SG and SCG. The 5-year survival rates of the SG, CG, and SCG were 69.3, 73.9, and 65.4 % in all stages, and 83.5, 82.2, and 70.4 % in stage I, respectively.

**Conclusions:**

Previous CVS did not increase the number of perioperative cardiovascular events in this study and had no significant influence on the prognosis of patients undergoing resection of NSCLC.

## Introduction

According to the latest annual report by the Japanese Association for Thoracic Surgery, approximately 64,000 cardiovascular surgeries (CVSs) and 36,000 surgeries for primary lung cancer are performed annually in Japan [1]. While recent long-term results following CVS have been favorable and general thoracic surgeons often perform lung cancer surgery after CVS, previous CVS is associated with problems during lung cancer surgery. First, some patients on antithrombotic therapy need to have their drugs suspended temporarily and be commenced on bridging anticoagulation therapy, indicating that they are at risk of bleeding and thrombosis. Second, adhesion in the pleural cavity from the previous CVS may result in severe intraoperative complications including bypass graft injury. Third, the risk of perioperative cardiovascular events in these patients remains unclear. In the present study, we used a thoracic revised cardiac risk index (ThRCRI), proposed by Brunelli et al. and Ferguson et al. [2–6], as a risk assessment tool for major cardiovascular events in the perioperative period. As there is no clear mechanism of how previous CVS affects the long-term results of non-small cell lung cancer (NSCLC) surgery, we investigated the surgical outcomes and long-term results of NSCLC resection in patients with a history of CVS.

## Patients and methods

### Patient groups

This retrospective study was based on the medical records at a single institute. We reviewed the clinical data of patients who underwent lung resection for NSCLC at our institute between January, 2003 and December, 2014 (*n* = 1138). Patients who underwent lung resection were divided into three groups: Those who had a history of CVS were classified as the study group (SG); those who did not have a history of CVS were classified as the control group (CG); and those with cardiovascular diseases but no history of CVS were classified as the specified control group (SCG). Cardiovascular diseases in the SCG included coronary artery disease (*n* = 93), valvular disease (*n* = 9), aortic aneurysm (*n* = 13), cardiomyopathy (*n* = 11), and arrhythmia (*n* = 42). Coronary artery disease (CAD) was treated with percutaneous coronary intervention (PCI) or medication, and the other diseases were controlled with medication. This study compared the surgical outcomes of the SG with those of the CG and SCG. The postoperative observation period was defined as the period from the operation to the last follow-up appointment.

### Preoperative evaluation of cardiac function

Preoperative resting electrocardiogram (ECG) and ultrasound echocardiography (UCG) were performed in all patients. Brain natriuretic peptide (BNP) was measured according to advice by the surgeon. Coronary angiography (CAG) was performed in patients with a history of a coronary artery bypass graft (CABG) or CAD, if recommended by cardiologists.

### Management of antithrombotic agents

The discontinuation of antithrombotic agents or the initiation of bridging therapy was decided by cardiologists. Antiplatelet agents were ceased 7 days before surgery, with warfarin discontinued 5–7 days before surgery and replaced by therapeutic bridging with unfractionated heparin (UFH). UFH was continued until 4–6 h before surgery and resumed as soon as possible after confirming no postoperative bleeding. The oral agents were resumed after postoperative day 1, and UFH was continued until the blood concentration or prothrombin time-international ratio was within the therapeutic range.

### Risk assessment for a postoperative major cardiac event

This study used ThRCRI to assess the risk of a perioperative major cardiovascular event. Postoperative major cardiovascular complications were defined as those occurring during admission or within 30 days after surgery. They included myocardial infarction (MI), pulmonary edema, ventricular arrhythmia requiring intervention or primary cardiac arrest, complete heart block, or any cardiac-related death [3, 4]. ThRCRI is a four-class risk score that includes four weighed factors: ischemic heart disease (IHD), defined as a history of MI, a positive exercise test, current complaint of chest pain considered to be related to myocardial ischemia, use of nitrate therapy, or an electrocardiogram with pathologic *Q* waves (score 1.5 points); cerebrovascular disease history, defined as a transient ischemic attack or stroke (score 1.5 points); a serum creatinine level of greater than 2 mg/dL (score 1 point); and pneumonectomy (score 1.5 points) [6]. The four ThRCRI classes based on the scores were as follows: category A (0 points), category B (1–1.5 points), category C (2–2.5 points), and category D (>2.5 points) [4]. The risk of a major cardiovascular event increases from class A to D [2–5]. In scoring the factors (1), if patients who underwent CABG for IHD were not taking any medication, they hardly obtained 1.5 points. This study further compared the risk in patients who underwent a major lung resection (lobectomy, bilobectomy, and pneumonectomy) between the two sets of two groups; namely, SG vs. CG and SG vs. SCG.

### Operative approaches

The surgical approach was classified into three categories: complete video-assisted thoracoscopic surgery (VATS), hybrid VATS (hVATS), and open thoracotomy (OT). Complete VATS was performed through a three- or two-port with utility minithoracotomy under thoracoscopic view. hVATS was performed through a two-port with anterolateral minithoracotomy under thoracoscopic view and direct visualization.

### Lung cancer staging

Lung cancer staging was done in accordance with the seventh edition of the tumor node metastasis (TNM) classification of lung cancer, as proposed by the Union for International Cancer Control and the International Association for the Study of Lung Cancer, 2009. For those patients who underwent lung resection before 2009, staging was still matched to the criteria in the seventh edition of the TNM classification.

### Definition of a perioperative event

Intraoperative bleeding was defined as requiring a blood transfusion or conversion to hVATS or OT to control the bleeding. Complications that occurred within 30 days of surgery were considered as postoperative complications and included arrhythmia that required intervention, prolonged air leakage that required pleurodesis, or bleeding that required a blood transfusion or reoperation. We defined 30- and 90-day mortality as death within 30 and 90 days after surgery, including after discharge, respectively.

### Statistical analyses

Continuous data are presented as the median and the interquartile range (IQR), and categorical data are presented as numbers and percentages. For comparing two groups, continuous data were analyzed by the Mann–Whitney’s *U* test, and categorical data were analyzed by the Fischer’s exact test or Chi-square test. A forward stepwise multiple regression analysis was performed to elucidate the relationships among intraoperative blood loss as a dependent variable and the following factors as independent variables: the presence of adhesion caused by previous CVS, bridging therapy, operation time, operative approach, and operative method. Logarithmic transformation of the dependent variable was performed to normalize its distribution. The 5-year survival rate of patients between January, 2003 and December, 2013 was estimated by the Kaplan–Meier method and differences between the two sets of the two groups were analyzed by the log-rank test. Probability less than 0.05 (*p* < 0.05) was considered significant. All statistical analyses were performed with JMP Pro 12.1 statistical software (SAS Institute, Cary, NC, USA).

### Ethics

This study was approved by the Research Ethics Committee of the Tokyo Women’s Medical University, Tokyo, Japan (No. 3568).

## Results

During the study period, 36 patients with a history of CVS underwent lung resection for NSCLC at our hospital. Of these 36 patients, 30 (83.3 %) received antithrombotic therapy, 19 (52.8 %) of whom also required bridging therapy in the perioperative period. According to preoperative resting UCG, the median ejection fraction was 55.2 % (IQR 50–65.6 %), and the median fractional shortening was 29.5 % (IQR 25.5–37 %). None of the patients underwent stress echocardiography. Preoperative BNP was measured in 17 patients, and the median level of BNP was 98.2 pg/mL (IQR 64.6–181.3 pg/mL). CAG was performed in 11 patients with a CABG history. A new diagnosis of CD was made in five patients, and PCI was performed for graft stenosis in seven patients. The median duration between previous CVS and lung resection was 96 months (IQR 49–152 months). The median postoperative observation period was 34.5 months (IQR 12.5–63.3 months).

Table 1 summarizes the baseline characteristics of the patients. There were significant differences in age (*p* = 0.0283), gender (*p* = 0.0081), number of comorbidities (*p* = 0.0066), and number of patients who required bridging therapy (*p* < 0.0001) between the SG and CG; and the number of comorbidities between the SG and SCG (*p* < 0.0001). The ThRCRI class was significantly different between the SG and SCG (*p* < 0.0001). The most common ThRCRI classes were class A in the SG and CG, and class B in the SCG. Table 2 shows the different CVS procedures that the SG patients underwent. CABG was the most common procedure, followed by aortic valve replacement. Combined surgical procedures included CABG with Dor, and CABG with mitral valve replacement. Surgery for congenital heart disease included ventricular septal defect closure with aortic valve replacement and atrial septal defect (ASD) closure. Eight patients had adhesion in the pleural cavity from the previous CVS (Table 3). Graft replacement and ASD closure were performed via lateral thoracotomy, and the other procedures were performed via median sternotomy. All of the patients with CABG history had adhesions surrounding the internal thoracic artery (ITA) grafts. There were no injuries to the ITA grafts caused by the dissection of adhesions.

**Table 1:** Comparison of patient characteristics

Variables	Study group (SG)	Control group (CG)	Specified control group (SCG)	*p* value*	
				SG vs. CG	SG vs. SCG
Total number of patients	36	1102	147		
Age (years)	73 (66.8–77.3)	69 (62–75)	74 (68–78)	0.0283	0.55
Gender				0.0081	0.372
Male	31 (86.1)	714 (64.8)	117 (79.6)		
Female	5 (13.9)	388 (35.2)	30 (20.4)		
Clinical stage				0.514	0.803
I	31 (86.1)	801 (72.7)	114 (77.5)		
II	2 (5.6)	120 (10.9)	14 (9.5)		
III	3 (8.3)	150 (14.1)	17 (11.6)		
IV	0	26 (2.3)	2 (1.4)		
Number of comorbidity				0.0066	<0.0001
0	5 (13.9)	354 (32.1)	5 (3.4)		
1	22 (61.0)	359 (32.6)	25 (17)		
2	5 (13.9)	246 (22.3)	51 (34.7)		
3	2 (5.6)	104 (9.4)	45 (30.6)		
≥4	2 (5.6)	39 (3.6)	21 (14.3)		
ThRCRI class				0.632	<0.0001
A	22 (91.7)	526 (82.2)	25 (29.4)		
B	2 (8.3)	101 (15.8)	50 (58.8)		
C	0	5 (0.8)	7 (8.3)		
D	0	8 (1.2)	3 (3.5)		
Bridging therapy				<0.0001	0.584
+	19 (52.8)	158 (14.3)	62 (42.2)		
–	17 (47.2)	944 (85.7)	85 (57.8)		

*ThRCRI* thoracic revised cardiac risk index

* Based on Fischer’s exact test or Chi-square test (categorical variables), or Mann–Whitney’s *U* test (continuous variables). Continuous variables are expressed as median and interquartile range; categorical variables are expressed as a number and percentage

**Table 2:** Types of previous cardiovascular surgery

Operative method	*n* = 36
CABG	21
Valve replacement	6
Graft replacement of descending aorta	3
TAR	2
Combined operation	2
Congenital	2

*CABG* coronary artery bypass grafting, *TAR* total arch replacement

**Table 3:** Cases of adhesion due to previous cardiovascular surgery

Previous cardiovascular surgery	Locations of adhesion	Types of lung resection	Number of cases (*n* = 8)
CABG	Surroundings of ITA graft		5
	RITA	RUL	3
		RML	1
	LITA	LUL	1
Graft replacement for TAA	In front of left upper lobe	Left S6 segmentectomy	1
AVR	Total pleural adhesion	LLL	1
ASD closure	Total plural adhesion	RLL	1

*ASD* indicates atrial septum defect, *AVR* aortic valve replacement, *CABG* coronary artery bypass graft, *ITA* internal thoracic artery, *LITA* left internal thoracic artery, *LLL* left lower lobectomy, *LUL* left upper lobectomy, *RITA* right internal thoracic artery, *RLL* right lower lobectomy, *RML* right middle lobectomy, *RUL* right upper lobectomy, *TAA* thoracic aorta aneurysm

There were no significant differences between the two sets of two groups in surgical and clinical outcomes (Table 4). In the SG, 24 patients underwent a major resection, 12 underwent a sublobar resection, and none underwent pneumonectomy. Three intraoperative complications occurred in the SG. In two patients, bleeding from pulmonary artery necessitated the VATS procedure to be converted to thoracotomy. In one patient, the blood pressure dropped after the induction of anesthesia, but the cause of this could not be determined, as ECG showed no evidence of IHD or arrhythmia. The operative method was then converted from lobectomy to wedge resection to shorten the operative time and no other intraoperative event occurred. Table 5 outlines the postoperative complications in both groups. In the SG, postoperative arrhythmia (atrial flutter), bleeding, and chylothorax did not require reoperation and there was no incidence of thrombosis, ventricular arrhythmia, or acute coronary syndrome in this group. Cardiovascular complications occurred at incidences of 2.8 % (*n* = 1) in the SG and 4.1 % (*n* = 6) in the SCG. One patient from the CG and SCG suffered a major cardiovascular event related to ventricular tachycardia. The 30- and 90-day mortality rate in the SG was zero.

**Table 4:** Comparison of surgical and clinical outcomes

Variables	Study group (SG) (*n* = 36)	Control group (CG) (*n* = 1102)	Specified control group (SCG) (*n* = 147)	*p* value*	
				SG vs. CG	SG vs. SCG
Operative approach				0.172	0.375
Complete VATS	28 (77.8)	839 (76.1)	108 (73.5)		
Hybrid VATS	4 (11.1)	56 (5.1)	10 (6.8)		
Open thoracotomy	4 (11.1)	207 (18.8)	29 (19.7)		
Operative method				0.834	0.355
Wedge resection	6 (16.7)	171 (15.7)	33 (19.7)		
Segmentectomy	6 (16.7)	239 (21.9)	29 (22.5)		
Lobectomy	23 (65.3)	627 (57.6)	82 (55.8)		
Bilobectomy	1 (2.8)	29 (2.7)	3 (2)		
Pneumonectomy	0	23 (2.1)	0		
Operative time (min)	204 (152–280)	195 (145–248)	191 (133–257)	0.334	0.304
Blood loss (ml)	102.5 (10–323)	74 (15–213)	70 (10–419)	0.528	0.357
Intraoperative complication	3 (8.3)	42 (3.8)	3 (2)	0.171	0.246
Bleeding	2	39	2		
Others	1	3	1		
Duration of drainage (days)	4 (3–6)	3 (3-5)	4 (3–6)	0.220	0.725
Length of stay (days)	10 (8–12)	10 (7–13)	11 (8–14)	0.804	0.725
Postoperative complication	4 (11.1)	116 (10.5)	21 (14.3)	0.911	0.619
30-day mortality	0	6 (0.54)	3 (2)	1	1
90-day mortality	0	7 (0.64)	4 (2.2)	1	1
Pathology				0.300	0.82
Adenocarcinoma	23 (63.9)	822 (74.6)	98 (66.7)		
Squamous cell carcinoma	10 (27.8)	198 (18.0)	15 (10.2)		
Others	3 (8.3)	82 (7.4)	34 (23.1)		
Pathological stage				0.870	0.831
I	28 (77.8)	767 (69.6)	109 (74.2)		
II	3 (8.3)	138 (12.5)	19 (12.9)		
III	5 (13.9)	172 (15.6)	17 (11.5)		
IV	0	25 (2.3)	2 (1.4)		

*VATS* video-assisted thoracoscopic surgery

* Based on Fischer’s exact test or Chi-square test (categorical variables), or Mann–Whitney’s *U* test (continuous variables). Continuous variables are expressed as median and interquartile range; categorical variables are expressed as a number and percentage

**Table 5:** Details of postoperative complications in both groups

	Study group	Control group	Specified control group
Total number of complications	5	135	28
Cardiovascular			
Arrhythmia	1	8	5
CHF	0	3	1
Bleeding	1	5	1
Acute exacerbation of IP	1	14	2
Prolonged air leakage	1	50	7
Chylothorax	1	3	3
Others	0	52	9

*CHF* congestive heart failure, *IP* interstitial pneumonia

Forward stepwise multiple regression analysis showed that the operative time (standardized *β* = 0.641, *p* < 0.0001), hVATS, or OT (standardized *β* = 0.294, *p* = 0.0272) were correlated with the intraoperative blood loss. Adhesion caused by previous CVS and bridging therapy was uncorrelated with the intraoperative blood loss.

**Fig. 1:**
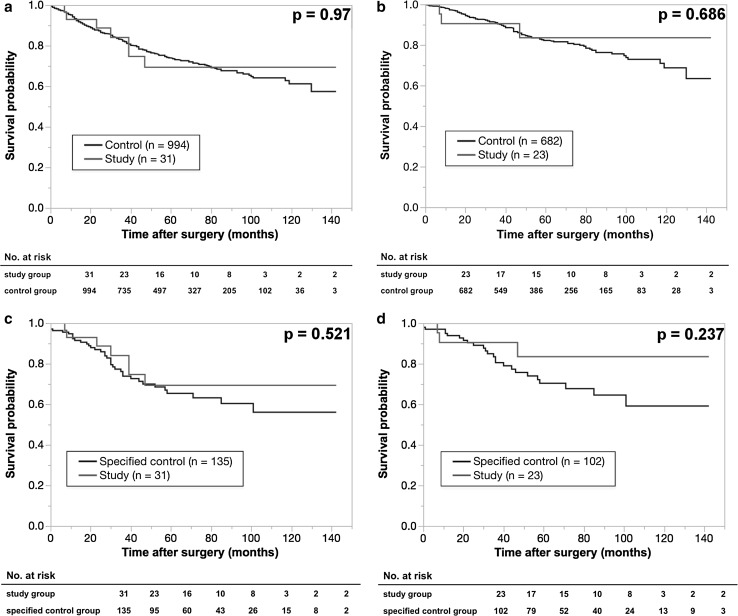
The survival curves of all stages and stage I of the two sets of two groups, which were the study group (SG) vs. the control group (CG), SG vs. the specified control group (SCG). **a** The 5-year survival rates of patients with all stages of lung cancer; 69.3 % in SG (*red line*, *n* = 31) and 73.9 % in CG (*blue line*, *n* = 994). **b** The 5-year survival rates of patients with stage I lung cancer; 83.5 % in SG (*red line*, *n* = 23) and 82.2 % in CG (*blue line*, *n* = 682). **c** The 5-year survival rates of patients with all stages of lung cancer; 69.3 % in SG (*red line*, *n* = 31) and 65.4 % in SCG (*blue line*, *n* = 135). **d** The 5-year survival rates of patients with stage I lung cancer; 83.5 % in SG (*red line*, *n* = 23) and 70.4 % in SCG (*blue line*, *n* = 102)

The 5-year overall survivals rates of patients with all stages vs. stage I were 69.3 and 83.5 % in the SG, 73.9 and 82.2 % in the CG, and 65.4 and 70.4 % in the SCG, respectively (Fig. 1). There were no significant differences between the two sets in two groups (all stages: *p* = 0.97, *p* = 0.521, stage I: *p* = 0.686, *p* = 0.237). In the SG, there were seven late deaths during the observation period; caused by lung cancer in six and CHF in one. The pathological staging results for the patients who died of lung cancer were stage IA (*n* = 2), IIB (*n* = 2), and IIIA (*n* = 2). Two patients with stage IA disease underwent limited resection because of poor cardiac function.

## Discussion

In this series of patients who underwent surgery for NSCLC, none of those with a history of CVS (SG) suffered severe cardiovascular complications in the perioperative period and their postoperative results were favorable, in spite of significant differences in age and the number of preoperative comorbidities from those of the control group (CG). On the other hand, those without a history of CVS, but with coexisting cardiac disease (SCG) had the highest incidence of postoperative cardiovascular complications. Although 52.8 % of the SG patients required bridging anticoagulant therapy, there were no significant differences in the duration of drainage or length of hospital stay from those of the CG. A forward stepwise multiple regression analysis showed that the bridging therapy and adhesion from previous CVS hardly influenced the intraoperative blood loss. Some guidelines on the management of antithrombotic agents in the perioperative period have been reported in Japan and Western countries [7–10]. While low-molecular weight heparin (LMWH) is generally used for bridging therapy in Western countries [7–9], UFH is used in Japan, because LMWH for bridging therapy is not covered by insurance. Although there are few differences in the time of resuming the agents after surgery, guidelines recommend that LMWH or UFH should be resumed after confirming no postoperative bleeding. This study also showed no significant thrombosis when adequate bridging therapy was given, even though these patients have a potential risk of thrombosis in the perioperative period. Furthermore, as postoperative atrial fibrillation (af), the most common arrhythmia after lung resection, can cause severe thrombosis, with sequela such as cerebral infarction, it is important to predict its occurrence in patients with previous CVS. Iwata et al. [11] reported that BNP and early transmitral velocity/tissue Doppler mitral annular early diastolic velocity in UCG findings may be risk factors for postoperative af. These preoperative examinations are commonly performed for patients with a history of CVS and may be useful predictors of postoperative af. Yamamoto et al. [12] also reported a high incidence of cerebral infarction in patients who undergo left upper lobectomy for lung cancer. However, the incidence of postoperative cerebral infarction after left upper lobectomy in patients with a history of CVS remains unclear and needs further investigation.

When thoracic surgeons perform lung resection in patients with a history of CVS, adhesions from their previous surgery can often make the resection more difficult. Adhesion between the ITA and the lung after CABG with an ITA graft must be dissected very carefully. To prevent graft injury, a technique was devised to leave the lung parenchyma on the graft, using a stapler [13, 14]. We applied this technique in two of the patients in this series, as we were concerned that their remnant lung parenchyma might become a source of infection. Shah et al. [13] reported that the incidence of pyothorax was higher in patients who underwent this technique than in a control group; however, there was no difference in cancer recurrence rates between two groups. If a graft injury can lead to fatal complications, this technique should be considered. In this study, patients who had undergone previous surgery via thoracotomy, including graft replacement for TAA or ASD closure, had extensive adhesions in the pleural cavity. VATS was very useful for providing good visualization to allow us to dissect the adhesions. In preparation for surgery, past operation records should be studied thoroughly to facilitate a safe surgical procedure.

According to an analysis of lung cancer registry cases in Japan, the rates of postoperative cardiovascular complications were 0.47 % for CHF, 0.07 % for MI, and 3.3 % for arrhythmia [15]. Thus, severe cardiovascular events rarely occur because of the progression of perioperative management and fortunately this study had no life-threatening events. Previous reports document the surgical results of patients undergoing lung cancer surgery with cardiovascular comorbidities [16–19]. Takenaka et al. [16] reported no increases in the postoperative morbidity and mortality rates and no influence on the long-term results, while Senbaklavaci et al. [20] reported that a CVS history hardly increased morbidity and mortality after major lung resection. In contrast, Ambrogi et al. [17] reported an increase in postoperative morbidity with a worse prognosis. Most thoracic surgeons will be concerned about the possibility of perioperative cardiovascular events in these patients. Brunelli et al. [2] proposed ThRCRI as a recalibrated version of the revised cardiac index. External validation in a single institute is verified [3, 4]: Ferguson et al. [5] reported the utility of a risk assessment of major postoperative cardiovascular events, referring to related cases from the Society of Thoracic Surgeons database. Brunelli et al. [6] reported that ThRCRI can be used to stratify the long-term prognosis after curative resection for stage I lung cancer. In our study, there was no significant difference in ThRCRI between the SG and the CG; however, when a risk assessment was done based on the ThRCRI, it was generally considered that patients with a history of CVS might be at higher risk of perioperative cardiovascular events because CAD and an aortic aneurysm are known to frequently coexist [21, 22]. CHF can develop in the long term in patients with a CVS history, and cardiovascular disease often develops in patients with systemic arteriosclerosis, diabetes mellitus, and chronic renal failure on hemodialysis. These are all risk factors for cerebrovascular disease and renal insufficiency and the four ThRCRI categories develop frequently in these patients. On the other hand, the most common ThRCRI class in the SCG in the present study was class B and there was a significant difference in the class of ThRCRI between the SG and the SCG because some patients who had undergone CABG for IHD were free from medications, they had no score factor, and 93 patients in the SCG had CAD, indicating that a higher ThRCRI score and another ThRCRI category were not uncommon in the SCG.

To maximize the chances of safe lung resection in patients with a history of CVS, their current cardiac condition must be assessed prior to performing the lung surgery. Therefore, we should expect that future lung resection after revascularization by CABG can be performed safely. Sergeant et al. reported that the percentages of patients who did not undergo any reintervention within 10 and 15 years after CABG were 89 and 72 %, respectively [23]. By several years post-CABG, the necessity for re-CABG and PCI may increase as a result of stenosis of the bypass graft and new CAD. This could apply to other types of CVS; for example, prosthetic valve dysfunction in the long term after valve surgery may result in heart failure and the need for reoperation, resulting in worsening cardiac function.

To our knowledge, there are no reports of long-term results following surgery for NSCLC in patients with a history of CVS. In Japan, the 5-year survival rate following resection of lung cancer is 70 % overall: 87 % for stage IA disease and 74 % for stage IB disease [24]. These results are consistent with those in the present study. The 5-year overall survival rate in the SG was not significantly different from those in the CG and the SCG. Although SG had a higher 5-year survival rate than the SCG for no clear reason, we speculate that the difference might have been affected by comorbidities other than lung cancer, because the number of comorbidities in the SCG was significantly higher than that in the SG. Although this study investigated a small number of cardiovascular events in a sample size of 36 patients, we found that previous CVS hardly affected the prognosis of the patients undergoing lung cancer resection. However, if patients undergo limited resection because of current poor cardiac function or severe adhesion, previous CVS may affect the NSCLC prognosis.

This study had some important limitations: First, it was a retrospective and single-institutional study and second, the SG had a smaller number of patients than the CG. Since only one patient suffered a major cardiovascular event appeared, a future study should be done on a greater number of patients. Third, no external validation of ThRCRI in Japanese patients has been performed. The types of previous CVS were heterogeneous, and an investigation of each type of past surgery should be considered in the future. Finally, cardiovascular diseases in the SCG were unable to be completely matched to cardiovascular diseases treated with surgery in the SG.

In conclusion, the adequate management of antithrombotic agents in the perioperative period allowed lung cancer resection to be performed without significant bleeding or thrombosis in patients with a history of CVS. This study showed that a history of CVS hardly increased the risk of a perioperative cardiovascular event and did not affect the prognosis of patients undergoing resection of NSCLC. However, limited resection due to the current cardiac status and the extent of adhesions may affect the prognosis of patients undergoing resection of NSCLC.
